# Thermal Behavior, Density and Viscosity of Terpene-Based Hydrophobic Eutectic Solvent Systems with Alcohols and Carboxylic Acids: Comparison with Tetrabutylphosphonium Bromide (TBPBr)-Based Systems

**DOI:** 10.3390/molecules31081336

**Published:** 2026-04-18

**Authors:** Jasmin Suljagić, Edita Bjelić, Mersiha Suljkanović, Snežana Papović, Janez Cerar, Milan Vraneš

**Affiliations:** 1 Faculty of Technology, University of Tuzla, Urfeta Vejzagića 8, 75000 Tuzla, Bosnia and Herzegovina; 2 Faculty of Natural Sciences and Mathematics, University of Tuzla, Urfeta Vejzagića 4, 75000 Tuzla, Bosnia and Herzegovina; 3 Faculty of Sciences, Department of Chemistry, Biochemistry and Environmental Protection, University of Novi Sad, Trg Dositeja Obradovića 3, 21000 Novi Sad, Serbia; 4 Faculty of Chemistry and Chemical Technology, University of Ljubljana, Večna pot 113, 1000 Ljubljana, Slovenia

**Keywords:** terpenes, TG/DSC, density, viscosity, Walden plot, ionicity

## Abstract

Hydrophobic eutectic solvent systems (ESSs) were prepared and characterized using temperature-dependent thermophysical and transport property measurements, supported by thermal analysis. The investigated systems comprise terpene-based mixtures, menthol:octanoic acid (1:2) and menthol:decanoic acid (1:1), and thymol-based mixtures, thymol:butanol (1:1), thymol:hexanol (1:1), thymol:octanoic acid (1:1), and thymol:oleic acid (1:1), as well as salt-containing ESSs based on tetrabutylphosphonium bromide (TBPBr), TBPBr:octanoic acid (1:1), and TBPBr:lauric acid (1:1). Density, dynamic viscosity, and electrical conductivity were measured at atmospheric pressure (*p* = 0.1 MPa) over 293.15–313.15 K. From density data, molar volumes and isobaric thermal expansion coefficients were calculated. The temperature dependence of viscosity was correlated with both Arrhenius and Vogel–Fulcher–Tammann equations. Conductivity results were used to compute molar conductivities, and the coupled conductivity–viscosity behavior was assessed via Walden analysis to quantify deviations from ideal electrolyte behavior and estimate ionicity. Thermal behavior and stability were evaluated by differential scanning calorimetry (DSC) and simultaneous thermogravimetric analysis (TG/DSC). The resulting dataset enables a consistent comparison of volumetric, flow, and ion transport descriptors across fully molecular terpene-based mixtures and TBPBr-containing systems. Overall, the combined transport descriptors, including Walden analysis, provide a practical framework for distinguishing molecular from salt-containing hydrophobic ESS families and support formulation selection for temperature-dependent applications, particularly in biphasic extraction processes.

## 1. Introduction

Replacing hazardous and volatile organic solvents remains a central goal of green chemistry and sustainable process design. In this context, several alternative solvent classes have been explored, including water-based systems, supercritical fluids, polymers, ionic liquids (ILs), and eutectic solvent mixtures [1]. Although ILs offer remarkable tunability and broad applicability, wider implementation is often limited by cost, synthesis and purification demands, and, for many structures, there are concerns related to persistence and toxicity [1].

Eutectic solvent systems are commonly described as mixtures of two or more components that form a stable liquid due to strong noncovalent interactions and non-ideal mixing, most prominently hydrogen bonding, complemented by dispersion and, in ionic systems, electrostatic contributions [2,3,4,5]. The literature also emphasizes that not all mixtures labeled as deep eutectic solvents are equally “deep” thermodynamically, and that rigorous classification would require solid–liquid equilibrium phase diagrams to locate true eutectic points and quantify deviations from ideality [4,5,6]. Since complete phase diagrams were not measured for all formulations in the present work, we adopt neutral terminology eutectic solvent systems (ESS) or eutectic mixtures throughout the manuscript.

Hydrophilic eutectic systems can be disadvantageous in aqueous process environments, where water uptake may cause compositional drift, dilution of the interaction network, and poor phase separation, which complicate extraction workflows and recycling [7,8]. These limitations have driven the development of hydrophobic eutectic liquids intentionally designed to be water-immiscible, thereby making them inherently suitable for biphasic systems [6]. Since hydrophobic eutectic systems were recognized as a distinct solvent class in the mid-2010s, the field has expanded rapidly, encompassing fundamental property studies as well as a broad range of applications, with extraction technologies and related separations dominating [6,9,10,11,12,13,14]. Beyond extraction, hydrophobic eutectic liquids have also been explored in electrochemical contexts, where hydrophobic media can provide distinct interfacial behavior and operational stability [15]. Across these uses, key design questions repeatedly arise, namely how composition controls density and viscosity and therefore mass transfer, how thermal stability and phase transitions constrain the working temperature window, and how reliably the liquid structure is maintained upon heating and cycling [4,6,9].

Two broad design strategies are commonly employed for hydrophobic eutectic systems. One uses hydrophobic ionic hydrogen bond acceptors, such as quaternary ammonium or phosphonium salts with hydrophobic alkyl substituents, paired with hydrophobic hydrogen bond donors, most often monocarboxylic acids or long-chain alcohols [6,9]. The second relies on entirely molecular mixtures assembled from naturally occurring and low-toxicity building blocks, such as terpenes and fatty acids, aligning with the natural eutectic solvent concept and being particularly attractive for food, pharmaceutical, and cosmetic applications [10,11]. Terpene-based systems, for example, menthol- or thymol-containing mixtures, provide hydrophobicity and moderate polarity tuning, while the acid or alcohol counterpart can modulate viscosity, density, and solvation capacity [11,16,17,18]. Importantly, performance is not determined solely by hydrophobicity. Intercomponent interactions, microheterogeneity, and the extent of ionization or ionic character can strongly influence transport properties and thermal behavior, which are critical for practical processing and recycling [4,6,9].

In this work, we systematically compare two families of hydrophobic ESS with distinct interaction motifs but similar water-immiscible character. The first family comprises terpene-based mixtures composed of neutral components, in which hydrogen bonding and dispersion interactions dominate. The second family comprises TBPBr-containing mixtures with fatty acids, in which the introduction of an ionic component adds Coulombic and ion–dipole contributions that can reshape volumetric response, flow resistance, and charge transport. Density, viscosity, and electrical conductivity were measured at atmospheric pressure over 293.15–313.15 K, and derived volumetric and transport descriptors were analyzed, including thermal expansion coefficients, Arrhenius and Vogel–Fulcher–Tammann correlations for viscosity, and Walden analysis for ionicity. Thermal behavior and practical stability limits were assessed by differential scanning calorimetry and thermogravimetric analysis. The resulting dataset enables consistent structure–property comparisons across fully molecular terpene-based mixtures and salt-containing systems, and provides reference data relevant to temperature-dependent applications, particularly biphasic extraction processes.

## 2. Results and Discussion

### 2.1. Density Results

**Figure 1:**
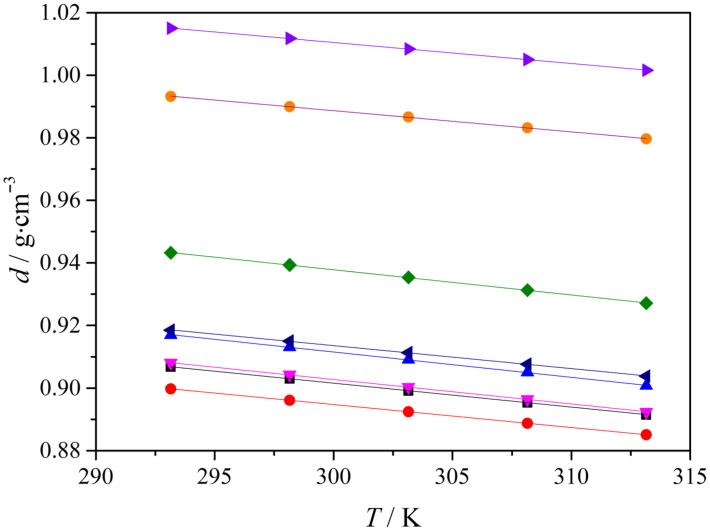
Density (*d*) as a function of temperature for the investigated hydrophobic eutectic solvent systems in the range 293.15–313.15 K at *p* = 0.1 MPa. Solid lines represent linear fits. Systems: (■) Men-OctA, (●) Men-DecA, (▲) Thy-but, (▼) Thy-hex, (♦) Thy-OctA, (◄) Thy-OleA, (►) TBPBr-OctA and (●) TBPBr-LauA.

The experimental density data for all investigated hydrophobic eutectic solvent systems are presented in Figure 1. In the studied temperature interval from 293.15 to 313.15 K, the density of each system decreases linearly with increasing temperature as described by Equation (1):

(1) $$d(g\cdot {\mathrm{cm}}^{-3})=b_{o}+b_{1}T(K)$$

In Equation (1), *d* is the density, and *T* is the absolute temperature. Parameters *b*_o_ and *b*_1_ were obtained by linear regression and are reported in Table 1.

The relative change in density (Δ*d*) is calculated using the following Equation (2):

(2) $$\Delta d(\%)=(d_{293.15K}-d_{313.15K})/d_{293.15K}\cdot 100$$

The linear fit coefficients and statistical parameters, including the correlation coefficient and the standard deviation of the fit, are presented in Table 1, confirming linear behavior across all mixtures in this range. Similar linear temperature dependences have been reported for terpene-based hydrophobic eutectic mixtures [11,16,17,18,19,20,21,22].

Ma et al. [23] measured the high-pressure densities of DL-menthol/octanoic acid eutectic solvents (at molar ratios including 1:1 and 1:2) over a broad temperature range (293.15–363.15 K) and found that densities decrease with increasing temperature, consistent with the trends observed in our hydrophobic mixtures. Their data show that the density of menthol/octanoic acid systems at atmospheric pressure and 293.15 K is in a similar range to the terpene-based eutectic densities reported here, and the temperature dependence follows a linear decline typical for these hydrophobic liquids. In the work by Deepika et al. [24], a series of hydrophobic DESs composed of menthol, thymol, and decanoic acid were characterized at atmospheric pressure in the temperature range between 293.15 and 353.15 K. They reported that densities for these menthol–thymol-based DESs decrease linearly with temperature and that the density increases with increasing thymol fraction in thymol–decanoic acid mixtures. Earlier work by Sas et al. [25] on DESs composed of menthol and various saturated fatty acids (octanoic, decanoic, dodecanoic) showed trends in density changes with temperature and composition that are consistent with our results. Their analysis at 293.15–413.15 K showed that longer chain acids tend to produce DESs with lower density at a given temperature, whereas mixtures rich in shorter acids or symmetric acid–acid combinations show higher densities.

The densities of TBPBr DESs decrease with increasing temperature but remain higher in absolute values than their ammonium analogs, due to the larger molar mass and ionic structuring of phosphonium-based systems [19,20].

The molar volume values (*V*_m_) of all ESSs are calculated using Equation (3):

(3) $$V_{m}({\mathrm{cm}}^{3}\cdot {\mathrm{mol}}^{-1})=M(g\cdot {\mathrm{mol}}^{-1})/d(g\cdot {\mathrm{cm}}^{-3}).$$

In Equation (3), *M* is the molar mass of the investigated ESSs, which is calculated as follows in Equation (4):

(4) $$M=x_{1}M_{1}+x_{2}M_{2}$$

In Equation (4), *x*_1_ and *x*_2_ are the mole fractions of components 1 and 2 as given in Table 1, while *M*_1_ and *M*_2_ are their molar masses. The calculated molar volume values of the investigated systems are reported in Table 2 together with the corresponding *M* values and the relative changes in molar volume (Δ*V*_m_), with increasing temperature from 293.15 to 313.15 K.

The isobaric thermal expansion coefficient values (*α*_p_) are also calculated using the following Equation (5):

(5) $$\alpha_{p}(K^{-1})=-1/d{\left(\partial d/\partial T\right)}_{p}.$$

**Figure 2:**
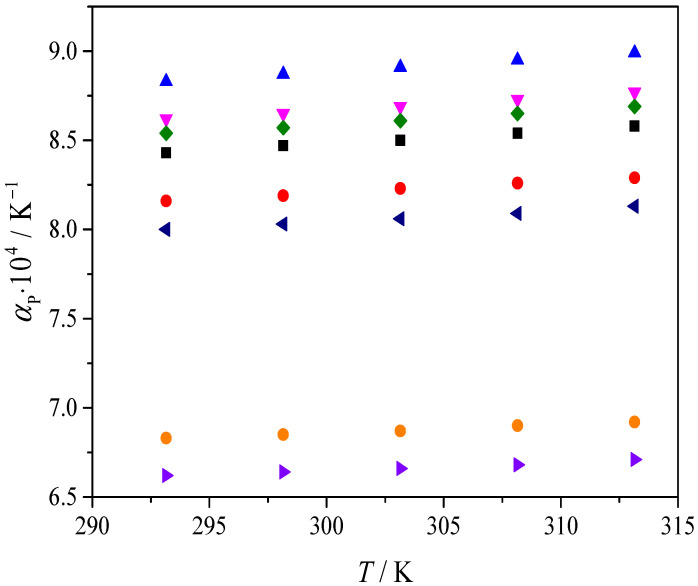
Dependence of the thermal expansion coefficient (*α*_p_) for investigated ESSs: (■) Men-OctA, (●) Men-DecA, (▲) Thy-but, (▼) Thy-hex, (♦) Thy-OctA, (◄) Thy-OleA, (►) TBPBr-OctA and (●) TBPBr-LauA.

The obtained results for the isobaric thermal expansion coefficient are presented in Figure 2.

The dependence of the isobaric thermal expansion coefficient on temperature in the range 293.15–313.15 K is shown in Figure 2.

A compositional trend is observed across the dataset. Increasing the carbon chain length of the alcohol or carboxylic acid component lowers density at all temperatures. This trend is seen in the thymol–alcohol series, where Thy-but has a higher density than Thy-hex at the same temperature. It is also seen when moving from thymol-octanoic acid to thymol–oleic acid, and in the TBPBr series, where TBPBr-OctA is denser than TBPBr-LauA, across the full temperature range. Similar chain-length effects on density have been reported for hydrophobic eutectic mixtures of thymol or menthol with monocarboxylic acids [11,16,17,18,21,22,26,27,28].

In addition to absolute density values, the relative change in density over 293.15–313.15 K provides a concise measure of temperature sensitivity. The terpene-based mixtures show larger relative decreases in density than the TBPBr-based mixtures over the same temperature interval. For example, the relative density decrease between 293.15 and 313.15 K is about 1.32–1.37% for TBPBr-based mixtures, while it is about 1.60–1.77% for terpene-based mixtures. A lower temperature sensitivity of density has also been observed for ESSs containing tetrabutylphosphonium bromide compared with related mixtures [19].

The separation between terpene-based and TBPBr-based systems can be understood in terms of dominant intermolecular interactions. In terpene-based mixtures, temperature-dependent packing and volume responses are primarily governed by hydrogen bonding between the neutral components, with dispersion interactions providing additional support. In TBPBr-based mixtures, the presence of ions introduces additional Coulombic contributions, including ion–ion interactions and ion-associated structuring, which can limit the extent to which thermal energy translates into volume increase over this temperature interval. This provides a consistent qualitative rationale for the smaller relative density changes observed in TBPBr-based systems.

When density is considered alongside molar volume, the same dataset becomes more informative. The calculated molar volumes increase with temperature across all systems, and the relative increase from 293.15 to 313.15 K is small yet systematic. At a given temperature, mixtures with longer alkyl chains exhibit larger molar volumes and therefore lower densities. This trend can be rationalized by a simple group-contribution argument. In homologous series, the molar volume often increases almost linearly with chain length, with an increment on the order of 16.4 cm^3^∙mol^−1^ per added methylene group [29], as reported for alkylpyridinium bromides from density-derived apparent molar volumes. In the present systems, a similar increase is observed in the molar volume differences. For example, the molar volume increases from 245.7 cm^3^∙mol^−1^ for Thy-but to 279.12 cm^3^∙mol^−1^ for Thy-hex, which corresponds to about 16.7 cm^3^∙mol^−1^ per added −CH_2_− group for the two additional methylene units. A similar estimate is obtained for TBPBr-OctA and TBPBr-LauA, where the molar volume difference of 67.2 cm^3^∙mol^−1^ corresponds to about 16.8 cm^3^∙mol^−1^ per −CH_2_− group for the four additional methylene units. Therefore, the density decrease with increasing chain length is consistent with the near-linear increase in molar volume upon adding −CH_2_− groups.

TBPBr-based mixtures exhibit higher densities than terpene-based mixtures at all temperatures. At *T* = 298.15 K, ESS TBPBr-OctA has a density of 1.011731 g∙cm^−3^, while the terpene-based systems are below 0.94 g∙cm^−3^. This difference is consistent with a higher average molar mass and a molar volume that is not proportionally larger. A useful illustration is the pair Thy-OleA and TBPBr-OctA. Their molar volumes are comparable across the measured range, yet TBPBr-OctA remains much denser, indicating that the larger mass contribution from the TBPBr component is not accompanied by a comparable increase in molar volume. Similar density levels for salt-containing ESSs relative to fully molecular eutectic mixtures are discussed in general deep eutectic systems and ESS reviews [4,6,24].

#### Isobaric Thermal Expansion Coefficient

The isobaric thermal expansion coefficient *α*_p_ is a practically important parameter because it quantifies how strongly a fluid’s volume changes with temperature, directly affecting volumetric stability during handling and storage, as well as in any application where temperature fluctuations can influence density-driven properties such as phase behavior, mass-to-volume dosing, and fluid power performance. In addition, *α*_p_ provides indirect insight into how intermolecular organization responds to heating, since larger *α*_p_ values indicate that thermal energy more effectively increases free volume and disrupts packing.

In the present study, *α*_p_ is obtained from the density–temperature dependence and is summarized with the temperature trend shown in Figure 2. For all systems, *α*_p_ increases slightly with temperature over 293.15–313.15 K, which indicates that the volumetric response becomes marginally more pronounced at higher temperatures in this interval. Terpene-based mixtures exhibit *α*_p_ values of about 8.0–9.0∙10^−4^ K^−1^, whereas TBPBr-based mixtures show lower values around 6.6–6.9∙10^−4^ K^−1^. The lower *α*_p_ values for TBPBr mixtures are consistent with a smaller increase in molar volume with temperature and a smaller relative density decrease in the same temperature range, indicating a less temperature-sensitive volumetric structure in the salt-containing systems.

From a molecular perspective, the difference in *α*_p_ can be attributed to the balance of dominant interactions. In terpene-based mixtures, heating primarily perturbs hydrogen bond connectivity and dispersion-controlled packing among neutral components, allowing thermal agitation to translate more efficiently into increased free-volume growth. In TBPBr-containing mixtures, electrostatic contributions, including ion–ion correlations and ion-associated structuring, impose stronger constraints on local organization, so the same temperature increase produces a smaller volumetric expansion. This interaction-based picture is consistent with general discussions of structural organization in hydrophobic ESS families, which differ in the presence or absence of an ionic component [4,6,7].

### 2.2. Viscosity Analysis

#### 2.2.1. Viscosity Results

The dynamic viscosities of the investigated ESSs at 293.15–313.15 K are summarized in Table 3. All systems show the expected decrease in viscosity with increasing temperature. However, absolute viscosity levels vary markedly across the dataset, reflecting differences in the strength and nature of intermolecular interactions and in the ease of structural rearrangement under shear. The fully molecular thymol–alcohol mixtures exhibit the lowest viscosities, whereas replacing the alcohol component with carboxylic acids yields higher viscosities, consistent with stronger associative interactions within the hydrophobic matrix. The TBPBr-based mixtures are the most viscous over the entire temperature interval, consistent with the presence of an ionic component that introduces electrostatic contributions and more persistent ion-associated structuring compared with neutral terpene-based mixtures. Viscosities show clear composition and temperature dependence. All systems decrease in viscosity with increasing temperature, but absolute levels differ markedly: thymol–alcohol mixtures exhibit the lowest viscosities, mixtures with carboxylic acids are more viscous due to stronger associative interactions, and TBPBr-based mixtures are the most viscous overall, reflecting the influence of the ionic component and persistent ion-associated structuring [19,20].

#### 2.2.2. Viscosity Flow Activation Parameters

The temperature dependence of viscosity in ESSs is most commonly described using Arrhenius and Vogel–Fulcher–Tammann (VFT) equations. Arrhenius-type correlations are often adequate for describing the viscosity of ESSs in low-to-moderate viscosity ranges and within limited temperature windows, whereas the VFT equation generally provides a more flexible description for highly viscous systems and over wider temperature ranges [30,31]. In view of this, the viscosity data in this work is correlated using both models. The parameters obtained from the Arrhenius and VFT fits should not be interpreted as directly equivalent activation energies. The Arrhenius treatment assumes a temperature-independent barrier and therefore provides a single average descriptor over the fitted interval, which is appropriate only when ln(*η*) is nearly linear with 1/*T*. In contrast, the VFT equation accounts for non-Arrhenius curvature and implies a temperature-dependent apparent barrier. Accordingly, larger discrepancies between Arrhenius- and VFT-based descriptors simply reflect more pronounced non-Arrhenius viscosity behavior in a given system, and VFT is expected to represent such systems more reliably over the investigated temperature range. The temperature dependence of viscosity was first analyzed using the Arrhenius-type relationship, per Equation (6):

(6) ln(η)=lnC+Ea/RT

where *C* is a constant, *R* is the gas constant, and *E*_a_ is the activation energy of viscous flow.

**Figure 3:**
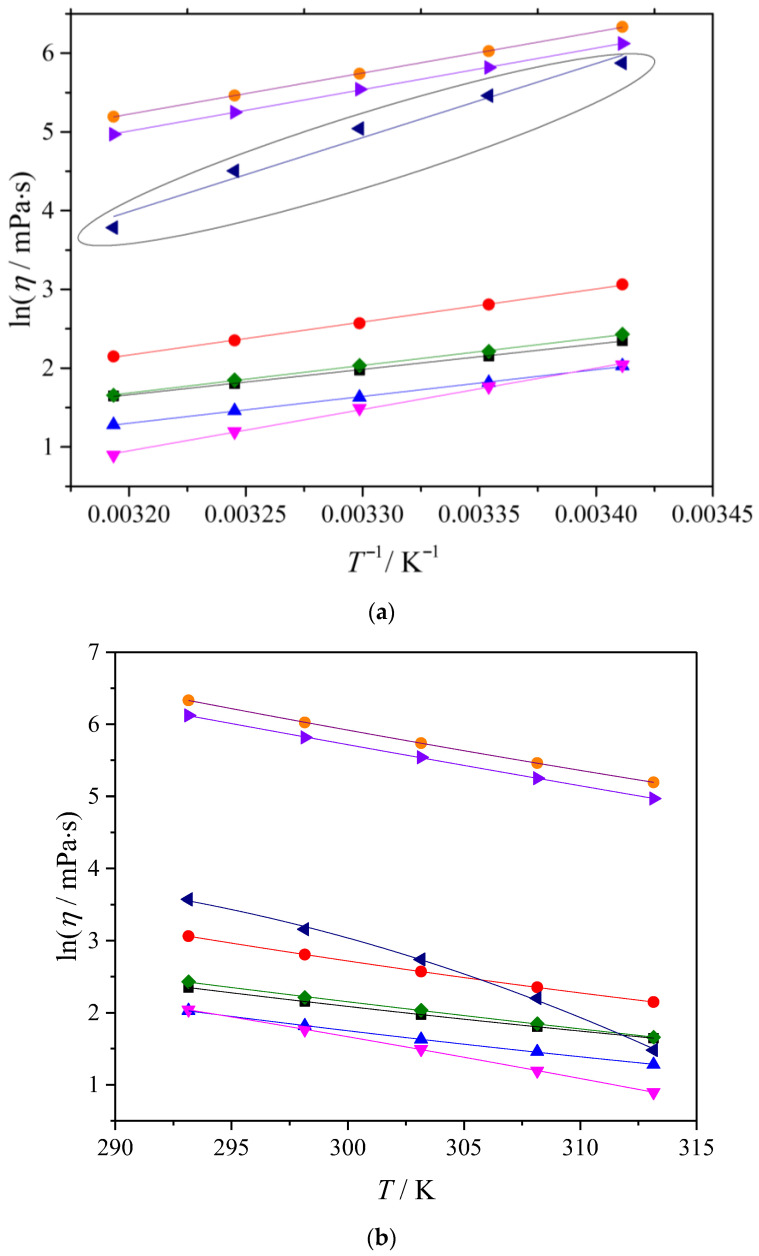
(**a**) Variation in ln(*η*) with *T*^−1^ and (**b**) variation in ln(*η*) with *T* for the investigated ESSs and variation of lnη for the investigated ESSs with T. Solid lines represent the best fits according to the VFT equation. Solid lines represent the best fits to the Arrhenius model. Systems: (■) Men-OctA, (●) Men-DecA, (▲) Thy-but, (▼) Thy-hex, (♦) Thy-OctA, (◄) Thy-OleA, (►) TBPBr-OctA and (●) TBPBr-LauA.

In the Arrhenius equation, the activation energy *E*_a_ is obtained from the slope of the ln(*η*) versus 1/*T* dependence. If Equation (6) is rewritten as *d*(ln(*η*))/*d*(1/*T*) = *E*_a_/*R*, it becomes clear that *E*_a_ is a compact descriptor of the degree of thermal activation of viscous flow over the investigated temperature interval. Higher *E*_a_ values indicate a stronger temperature dependence of viscosity in this interval, meaning that viscous flow requires overcoming larger transient structural constraints, whereas lower *E*_a_ values suggest easier rearrangement during shear and a weaker temperature dependence. The results of the Arrhenius fit are summarized in Table 4, together with the regression statistics (standard deviation and coefficient of determination), and the corresponding ln(*η*) versus 1/*T* plot is shown in Figure 3a.

As shown in Figure 3a and with the fit statistics in Table 4, the Arrhenius equation describes the viscosity–temperature dependence very well for all systems in the range 293.15–313.15 K. The only mixture with noticeably reduced fit quality is thymol–oleic acid, indicating a deviation from Arrhenius behavior. This suggests that, in this system, the flow mechanism is not constant across the interval.

The obtained *E*_a_ values reflect meaningful compositional differences. In fully molecular terpene-based systems, viscous flow is governed primarily by the disruption and reformation of hydrogen-bonded and dispersion-dominated contacts. In TBPBr-containing mixtures, the presence of an ionic component introduces additional electrostatic contributions and ion-associated structuring, which increase the energetic barrier to rearrangement under shear and are reflected in higher *E*_a_ values. This interpretation is consistent with the substantially higher viscosities of TBPBr-based mixtures observed in Table 3.

The viscosity–temperature dependence of the investigated ESSs was also correlated using the Vogel–Fulcher–Tammann equation, as Equation (7):

(7) $$\ln(\eta )=A+B/(T-T_{o})$$

where *A* and *B* are fitting constants, and *T*_o_ is the Vogel temperature. The VFT fits are shown in Figure 3b, and the obtained parameters, along with the regression statistical descriptors, are listed in Table 5. Compared with the Arrhenius approach, the VFT model allows for a non-linear temperature dependence of ln(*η*) and is therefore frequently used for ESSs and other associated liquids whose transport properties are influenced by temperature-dependent structural relaxation.

The activation energy can also be calculated from the VFT Equation (8) by transforming it as follows:

(8) $$E_{\eta }=RBT^{2}/{\left(T-T_{o}\right)}^{2}$$

The calculated activation energy values at *T* = 298.15 K (*E*_η(298.15K)_) are given in Table 5.

The VFT fits shown in Figure 3b provide high-quality correlations for all investigated mixtures, including the thymol–oleic acid system, which deviates from a strictly linear Arrhenius dependence. This indicates that, within the studied interval, the viscosity response is better represented by a model in which the apparent barrier to flow varies with temperature rather than remaining constant. The results for the ESS with an equimolar ratio of thymol and oleic acid are in good agreement with the VFT fits reported for the thymol–oleic acid system at a thymol-to-oleic acid molar ratio of 2:3 [17].

### 2.3. Electrical Conductivity

Because many ESSs are relatively viscous liquids, their electrical conductivities at room temperature are often below 1 mS·cm^−1^. The electrical conductivities of the investigated ESSs were measured at atmospheric pressure over the temperature range 293.15–313.15 K. The resulting conductivity data are summarized in Table 6. Overall, the results span several orders of magnitude and clearly distinguish the fully molecular terpene-based mixtures from the TBPBr-containing systems.

The thymol–alcohol mixtures show very low conductivities in the 10^−3^–10^−2^ μS·cm^−1^ range and only a modest increase with temperature. These values indicate that these liquids are essentially non-ionic, and the measured conductivity is most plausibly attributed to trace ionic impurities and, to a very limited extent, acid–base equilibria, for example, a weak dissociation of the phenolic OH group of thymol, rather than to a significant concentration of mobile charge carriers. In contrast, the terpene–carboxylic acid mixtures exhibit higher, yet still very low, conductivities in the sub-µS·cm^−1^ range and show a more pronounced increase with temperature. This increase relative to the thymol–alcohol systems is consistent with the presence of a carboxylic acid component, which increases the overall polarity of the mixture and can generate a small population of ionic species compared with the predominantly neutral alcohol-based mixtures.

The TBPBr-based ESSs exhibit conductivities that are orders of magnitude higher than those of the terpene-based systems and increase strongly with temperature. This behavior directly reflects the presence of an ionic component, so the conductivity is governed by ion mobility and its temperature dependence. The increase in *κ* with temperature is consistent with reduced viscous resistance and enhanced ion transport as the liquid becomes less viscous, and it may also reflect changes in ion association with temperature. Importantly, this is the clearest experimental indicator in the dataset that the TBPBr-containing mixtures possess markedly higher ionic character than the fully molecular terpene-based ESSs.

### 2.4. Conductivity–Viscosity Correlation: Walden Plot and Ionicity

Angell and co-workers [32,33,34] propose the Walden plot as a practical tool for evaluating ionicity and ion–ion interactions by correlating the logarithm of molar conductivity, log(*Λ*) in S·cm^2^·mol^−1^, with the logarithm of fluidity, log(1/*η*(Poise)). Molar conductivity values (*Λ*) for the measured systems are calculated from the measured electrical conductivity and density data using Equation (9):

(9) Λ=κM/d,

where *Λ* is the molar conductivity, *κ* is the electrical conductivity, *M* is the molar mass, and *d* is the density of the investigated ESS, and *Λ* are presented in Table 7.

**Figure 4:**
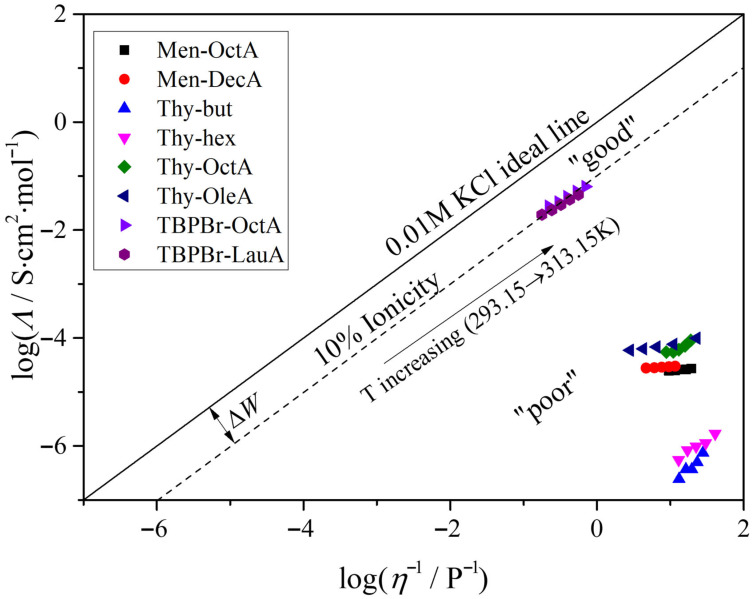
Walden plot.

The concept originates in the Walden rule for electrolyte solutions, which relates ionic transport to viscous resistance and predicts an approximately linear relationship between *Λ* and 1/*η* when the number of charge carriers is essentially constant. For strong electrolytes that are effectively fully dissociated in dilute aqueous solution, such as 0.01 M KCl, the temperature dependence of molar conductivity is governed predominantly by changes in ionic mobility and therefore closely follows the temperature dependence of viscosity. In contrast, for weak electrolytes, the degree of dissociation can vary with temperature, so *Λ* is influenced not only by mobility but also by changes in the concentration of charge carriers. Consequently, deviations of a weak electrolyte from the 0.01 M KCl reference at the same temperature and fluidity can serve as a useful indicator of reduced ionicity and incomplete dissociation. The degree of deviation from the ideal 0.01 M KCl behavior is presented graphically in the Walden plot shown in Figure 4. All investigated ESSs fall below the 0.01 M KCl line, indicating non-ideal charge transport relative to the fully dissociated aqueous reference.

A clear separation is evident between the fully molecular terpene-based mixtures and the TBPBr-containing systems. The terpene-based ESSs lie well below the reference line, consistent with their extremely low molar conductivities and indicating that these liquids contain only a very small population of mobile charge carriers. Within this group, the thymol–alcohol systems occupy the lowest region of the plot, while terpene–carboxylic acid mixtures are shifted slightly upward, in agreement with their higher conductivities, yet still remain far from the ideal line. In contrast, the TBPBr-based mixtures are much closer to the 0.01 M KCl line, reflecting their substantially higher ionic character and indicating that electrical transport is dominated by ionic species whose mobility is strongly coupled to viscosity.

As temperature increases, the data points shift toward higher fluidity and molar conductivity, consistent with reduced viscous resistance and enhanced ion mobility. The relative placement of each ESS family on the Walden plot therefore provides a compact visualization of the transport regime: strongly sub-ideal behavior for fully molecular terpene systems and markedly less sub-ideal behavior for TBPBr-containing mixtures, in which ionic contributions to charge transport remain significant even in a hydrophobic matrix.

To quantify the deviation from the ideal 0.01 M KCl behavior, the ionicity (%) can be calculated using Equations (10) and (11):

(10) $$\Delta W=\log\eta^{-1}-\log\Lambda ,$$

(11) $${\%\mathrm{Ionicity}=10}^{-\Delta W}\cdot 100.$$

The calculated ionicity values are presented in Table 8.

On the ionicity scale commonly adopted in the literature, Δ*W* = 0 corresponds to 100% ionicity, while Δ*W* = 1 corresponds to 10% ionicity, meaning that the liquid exhibits only 10% of the ideal molar conductivity expected at the same viscous resistance (see Figure 4). In the Angell-type classification based on the Walden plot, electrolytes located close to the KCl reference are therefore considered “good” ionic systems, whereas those falling below the 10% line are described as “poor” ionic systems because their molar conductivity is strongly suppressed relative to the ideal expectation at comparable fluidity.

The terpene-based ESSs exhibit ionicity values of 10^−6^–10^−3^%, consistent with essentially non-ionic transport and indicating that the measured conductivities are dominated by trace charge carriers rather than by a meaningful concentration of mobile ions. In contrast, the TBPBr-based mixtures show ionicity values of approximately 8–13%, placing them near, yet below, the 10% line and classifying them as “intermediate” between “good” and “poor ionic” systems in the Angell-type interpretation. This indicates that charge transport in these liquids is governed by ionic species but remains substantially suppressed relative to the ideal KCl reference due to persistent ion–ion correlations and partial ion association in the hydrophobic matrix.

In this study, the Walden plot is also applicable to hydrophobic systems, clearly distinguishing three transport regimes: essentially non-ionic terpene–alcohol mixtures, terpene–carboxylic acid mixtures with limited ion generation, and salt-containing TBPBr-based ESSs with predominantly ionic charge transport.

### 2.5. Thermal Measurements

#### 2.5.1. Thermogravimetric (TG) Measurements

**Figure 5:**
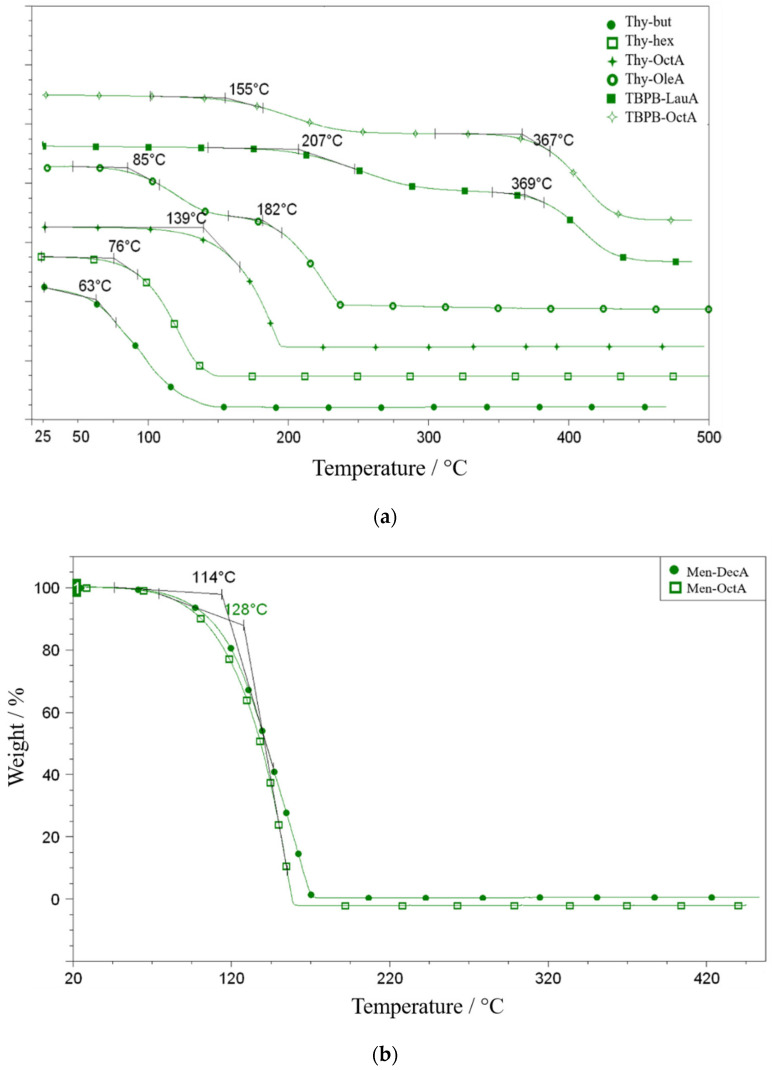
Thermogram for the investigated ESSs based on: (**a**) thymol and tetrabutylphosphonium bromide, and (**b**) menthol.The curves were vertically shifted to avoid overlap.

The thermal behavior of the investigated ESSs was assessed by simultaneous TG, DTG, and DSC under a nitrogen atmosphere. Because several systems contain volatile neutral components, such as alcohols and terpenes, and the measurements were performed in an open pan, the initial mass loss observed in TG is interpreted primarily as volatilization rather than true chemical decomposition [35]. The temperatures discussed below, therefore, refer to the onset of measurable mass loss *T*_onset_ and serve as practical descriptors of the upper working temperature window under open conditions. Figure 5 shows representative thermograms for the main ESS families.

Thymol–alcohol mixtures show the earliest mass loss, consistent with the volatility of the alcohol component, as shown in Figure 5. The thy-but system exhibits a *T*_onset_ of about 63 °C, and Thy-hex shows a *T*_onset_ of about 76 °C. These onsets occur well below the boiling points of the pure alcohols, since 1-butanol boils at about 118 °C and 1-hexanol at about 157 °C. This comparison supports a volatilization-dominated first TG step and indicates that mixing does not suppress evaporation of the more volatile constituent under the applied open-pan TG conditions. Although thymol can form hydrogen-bonding interactions with the alcohol oxygen atom [36], the TG response suggests that these interactions do not prevent the early loss of the alcohol-rich fraction under dynamic heating and nitrogen flow. From an application perspective, these mixtures are therefore better suited for low-temperature use and for closed or well-sealed systems, where compositional changes due to evaporation are minimized.

In thymol–acid systems, the onset of mass loss shifts to higher temperatures relative to thymol–alcohol mixtures, mainly due to the lower volatility of the acid component, with possible additional contributions from specific intermolecular interactions in the liquid phase [37]. Thy-OctA shows a *T*_onset_ of about 139 °C in Figure 5. The Thy-OleA system exhibits a two-step mass loss profile, with *T*_onset1_ of about 85 °C followed by a second event around 182 °C, suggesting sequential removal of a more volatile fraction and subsequent thermal transformations of the remaining matrix. The first TG step occurs well below the boiling points of the corresponding pure acids; for example, octanoic acid boils near 239 °C and lauric acid near 298 °C, which again supports volatilization and co-volatilization as the dominant origin of low-temperature mass loss in open-pan TG experiments.

Menthol-based ESSs in Figure 5b show intermediate *T*_onset_ values, with about 114 °C for Men-OctA and about 128 °C for Men-DecA. The increase with acid chain length is consistent with reduced volatility and stronger dispersion interactions in the menthol–acid matrix [38,39]. Terpene–fatty acid ESSs offer a broader usable temperature window and a lower risk of rapid composition drift than terpene alcohol mixtures, making them more suitable for applications involving moderate heating or prolonged operation. Table 9 also includes the values of the decomposition temperatures, melting temperatures and crystallization temperatures of all investigated systems.

TBPBr-based mixtures exhibit the highest thermal resistance and a characteristic two-step TG profile shown in Figure 5a. TBPBr-OctA shows *T*_onset1_ of about 155 °C, followed by a high-temperature event around 367 °C, whereas TBPBr-LauA shows *T*_onset1_ of about 207 °C and a second event around 369 °C. The lower-temperature step can be attributed to the loss of a more volatile or weakly bound fraction, while the high-temperature step reflects decomposition of the ionic residue [40]. Compared with terpene-only systems, the presence of the salt markedly expands the thermal stability window.

**Table 9:** Decomposition temperature (*T*_decomp_), temperature of melting (*T*_melt_), values of the crystallization temperature (*T*_cryst_) of the investigated systems in this work and found in the literature.

System	*T*_decomp_ (°C)	*T*_melt_ (°C)	*T*_cryst_ (°C)
Thy-OctA	139	−2.4	−6.98
Thy-OleA	85, 182	0.5	−30.9
Thymol	150	49.5 [36]	50
OleA	200	14 [41]	4
OctA	239	16 [42]	15
TBPBr-OctA	155, 367	1.5	/
TBPBr-LauricA	207, 369	5.5	−22.6
LauricA	298	43 [42]	40
TBPBr	210	100 [43]	40
Thy-But	63	/	/
Thy-Hex	76	/	/
Butanol	118	/	/
Hexanol	157	/	/
Ment-DecA	128	9	−10
Menthol	165	41 [44]	−5
DecA	125	31.5 [45]	30
Ment-OctA	114	−11	−21

#### 2.5.2. Differential Scanning Calorimetry (DSC) Measurements

**Figure 6:**
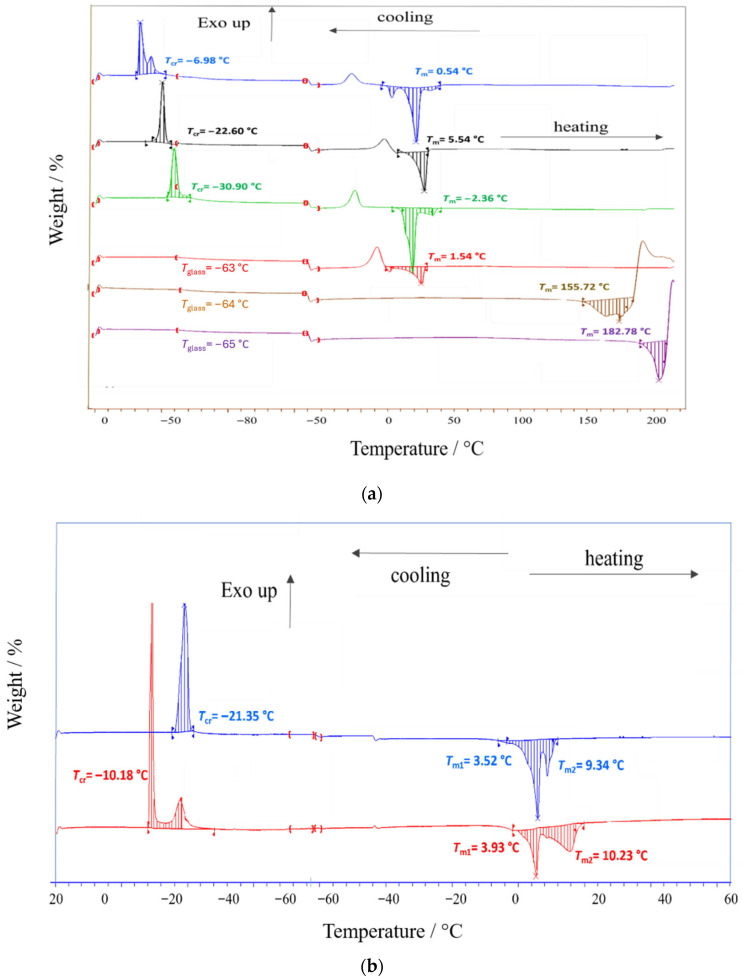
(**a**) Differential scanning calorimetry (DSC) curves of ESSs for: (**––**) Thy-OleA, (**––**) TBPBr-LauricA, (**––**) Thy-OctA, (**––**) TBPBr-OctA, (**––**) Thy-But and (**––**) Thy-Hex; (**b**) eutectic solvents for: (**––**) Ment-DecA and (**––**) Ment-OctA.

Differential scanning calorimetry was used to identify phase transitions in the investigated ESSs, primarily the melting temperature (*T*_m_) and the crystallization temperature (*T*_cr_). Notably, DSC measurements were performed in hermetically sealed pans, whereas TG experiments were carried out in open pans. Therefore, DSC captures thermal events of a composition that remains effectively constant during heating, whereas TG reflects practical mass loss under open conditions, which can be dominated by the evaporation of volatile constituents. This methodological difference is particularly important for alcohol-containing systems and explains why high-temperature endothermic features can be observed in sealed DSC traces even when TG indicates early mass loss under open conditions. Figure 6 summarizes DSC curves for thymol-based and TBPBr-based systems, while Figure 6b shows DSC curves for menthol-based systems.

For thymol-based mixtures with fatty acids, the measured melting points are markedly lower than those of the neat constituents, confirming strong melting-point depression and eutectic-like behavior. Literature reports the melting temperature of thymol as 49.5 °C [36]. The melting temperatures of oleic acid and octanoic acid are reported as 14 °C [41] and 16 °C [42], respectively. In the present work, Thy-OleA melts at about 0.5 °C, while Thy-OctA melts at about −2.4 °C. These values demonstrate that mixing thymol with fatty acids substantially stabilizes the liquid state at low temperatures, which can be attributed to the disruption of lattice packing and the stabilization of the liquid phase by specific interactions, including hydrogen bonding [37,43]. The DSC curve shapes are similar for Thy-OleA and Thy-OctA, indicating comparable melting behavior within this family. Upon cooling, crystallization occurs at about −7 °C for Thy-OleA and at about −31 °C for Thy-OctA. The much lower *T*_cr_ for Thy-OctA indicates stronger suppression of crystallization under the applied cooling protocol, consistent with slower nucleation and growth or more frustrated packing in the octanoate-based mixture.

A similar melting point depression is observed in TBPBr-based mixtures with fatty acids. The literature reports the melting temperature of tetrabutylphosphonium bromide as 100 °C [40]. The literature melting temperatures of lauric acid and octanoic acid are 43 °C [42] and 16 °C [42], respectively. In contrast, the melting points of the prepared mixtures are 5.5 °C for TBPBr-LauA and 1.5 °C for TBPBr-OctA. During cooling, TBPBr-LauA crystallizes at about −22.6 °C, whereas a distinct crystallization peak is not observed for TBPBr-OctA within the scanned range. This behavior suggests that crystallization is more effectively suppressed in the octanoate-based system, likely due to slower nucleation kinetics or kinetic trapping at the applied cooling rate.

For thymol–alcohol mixtures, Figure 6 shows high-temperature endothermic features at approximately 156 °C for Thy-but and 183 °C for Thy-hex. These events should not be described as thermal degradation without additional evidence. Given the volatile nature of alcohol-containing mixtures and the use of hermetically sealed pans, the observed DSC signals are more consistently attributed to evaporation-related processes, pressure-driven reequilibration within the closed system, and irreversible thermal changes that do not correspond to equilibrium melting and crystallization transitions at the applied thermal treatment. The phase changes as glass transition (*T*_glass_) appear at low-phase transition temperatures, as presented at Figure 6 with a selected part indicating a very slight phase transition.

Menthol-based mixtures shown in Figure 6b exhibit two endothermic peaks during heating, indicating melting over two temperature regions and suggesting more complex solid-state organization or multiple domains that melt over different intervals. The main melting temperatures are about −11 °C for Ment-OctA and about 9 °C for Ment-DecA. Crystallization occurs at about −21 °C for Ment-OctA and at about −10 °C for Ment-DecA. Therefore, the crystallization temperature shifts to a higher value for the Ment-DecA system, indicating that crystallization is easier under the applied conditions than for Ment-OctA. This trend is consistent with the higher melting temperature of Ment-DecA and reflects composition-dependent packing effects in menthol fatty acid matrices. The literature reports the melting temperature of menthol in the range from 41 to 44 °C depending on purity [44], while decanoic acid melts at 31.5 °C [45]. The much lower melting temperatures of the mixtures again confirm pronounced melting point depression and stabilization of the liquid phase. The overall shapes of the DSC curves agree with previously reported data for similar menthol-based systems [11,45,46,47,48].

## 3. Experimental Section

### 3.1. Preparation Procedure for the Eutectic Solvent Systems

All reagents were used as received without further purification, and detailed specifications are provided in Table 10. Hydrophobic eutectic solvent systems (ESSs) were prepared by mixing the selected components in the appropriate molar ratios. The appropriate amounts were weighed into a sealed glass vial and heated under stirring until a clear and homogeneous liquid formed. The samples were then allowed to cool to room temperature and were visually inspected. A stable ESS was confirmed when the sample remained a single-phase transparent liquid after 24 h of storage under ambient conditions. All ESSs were stored in tightly closed vials prior to further measurements.

The list of the prepared ESSs, their component molar ratios, and the corresponding abbreviations are presented in Table 11.

### 3.2. Apparatus and Procedures

#### 3.2.1. Thermogravimetric Analysis

Thermogravimetric analysis was performed using a TG thermal analyzer SDT Q600 (TA Instruments, New Castle, DE, USA). Approximately 3.0 mg of each eutectic solvent system was placed in an open platinum pan. Measurements were carried out under a nitrogen atmosphere with a purge flow rate of 100 cm^3^·min^−1^ from room temperature to 500 °C at a heating rate of 10 °C·min^−1^. Temperature calibration was performed over the range 25–1000 °C using the Curie point of a nickel standard with open platinum crucibles and a dry nitrogen purge flow of 100 cm^3^·min^−1^.

#### 3.2.2. Differential Scanning Calorimetry

Differential scanning calorimetry (DSC) measurements were performed on a Mettler Toledo DSC1 instrument. The sample was first cooled from room temperature (25 °C) to −80 °C at a cooling rate of 10 °C·min^−1^ and held for 5 min. It was then heated to 200 °C at a heating rate of 10 °C·min^−1^. For the determination of the melting points of various eutectic solvent systems based on thymol, approximately 4 mg of sample was precisely weighed into a 40 µL aluminum crucible on a Mettler Toledo MX5 balance and hermetically sealed. Temperature and enthalpy were calibrated using indium and zinc standards supplied by Mettler Toledo. An empty crucible served as a reference.

#### 3.2.3. Density Measurements

Density measurements were performed using an Anton Paar DSA 5000 M density and sound velocity analyzer (Anton Paar, Graz, Austria) based on the oscillating U-tube principle. In this work, only density data are reported. The density accuracy of the instrument is ±0.000005 g·cm^−3^. Prior to the measurements and after each sample, the calibration was verified by measuring the density of distilled water in the temperature range 293.15–313.15 K. At each temperature, measurements were performed in triplicate, and the mean values are reported.

#### 3.2.4. Viscosity Measurements

Dynamic viscosity was measured as a function of temperature from 293.15 to 313.15 K at atmospheric pressure. For each sample at each temperature, viscosity was recorded in triplicate and the mean values are reported. The low- to medium-viscosity systems Men–OctA, Men–DecA, Thy–but, Thy–hex, and Thy–OctA were measured with an Anton Paar Lovis 2000 M/ME rolling-ball viscometer equipped with an integrated Peltier thermostat, providing temperature control with a repeatability (s.d.) of 0.005 °C and an accuracy of 0.02 °C. Viscosity measurements with this instrument have an accuracy of 0.5% and a repeatability of 0.1%. The highly viscous systems Thy–OleA, TBPBr–OctA, and TBPBr–LauA were also measured with a Brookfield DV-II+ Pro viscometer using an SC4-18 spindle. The measurement cell was connected to a Lauda E100 thermostatic bath, which provided temperature stability within ±0.01 K. The rotational speed was adjusted to achieve an appropriate torque range (50–200 RPM). Viscosity data were recorded and further processed using the software package Origin 2016.

#### 3.2.5. Electrical Conductivity Measurements

Electrical conductivity measurements were performed at atmospheric pressure using a Mettler Toledo SevenExcellence S470 conductivity meter equipped with a suitable conductivity cell. The instrument covers a conductivity range from 0.001 μS·cm^−1^ to 2000 mS·cm^−1^, with a resolution of 0.001–2 and an accuracy of ±0.5%. The temperature of the conductivity cell was maintained with an external circulating thermostat with a stability of ±0.01 K. Prior to measurements, the instrument was calibrated with certified KCl standard solutions supplied by the manufacturer. Conductivity was measured over the temperature range 293.15–313.15 K after thermal equilibration at each temperature. At each temperature, conductivity was recorded in triplicate and the mean values are reported.

## 4. Conclusions

This study provides a consistent thermophysical and thermal dataset for two families of hydrophobic eutectic solvent systems (ESS), namely terpene-based molecular mixtures and TBPBr–fatty acid systems. Density, viscosity, and electrical conductivity were measured over 293.15–313.15 K, and combined with TG and DSC to define volumetric, flow, ion-transport, and thermal descriptors relevant to practical use.

All systems exhibit a linear decrease in density with temperature, enabling reliable calculation of molar volumes and isobaric thermal expansion coefficients. Increasing the alkyl-chain length of the alcohol or acid component lowers density and increases molar volume, whereas TBPBr-containing systems remain denser and show lower thermal expansivity. Viscosity decreases strongly with temperature for all mixtures, with the lowest values in thymol–alcohol systems and the highest in TBPBr-based systems.

Electrical conductivity and Walden analysis provide the clearest separation between the two ESS families. Terpene-based mixtures show negligible ionicity and essentially non-ionic transport, while TBPBr-containing mixtures display much higher conductivities and measurable ionicity, indicating predominantly ionic transport that remains sub-ideal due to ion association in the hydrophobic matrix. TG and DSC further delimit practical operating windows and demonstrate that thermal behavior depends on constituent volatility and on acid or salt pairing.

Overall, the results show that introducing an ionic component markedly reshapes volumetric response, flow resistance, and charge transport even under hydrophobic conditions. The reported trends and reference data support rational selection of hydrophobic ESS formulations for temperature-dependent applications, particularly biphasic extraction processes where viscosity, phase handling, and thermal limits must be balanced.

## Figures and Tables

**Table 1:** Linear regression parameters for the density–temperature correlation (Equation (1)), together with the correlation coefficient (*R*^2^) and the standard deviation (*σ*) of the fit for the investigated ESSs in the range 293.15–313.15 K.

SYSTEM	*b*_o_/(g·cm^−3^)	*b*_1_ × 10^4^/(g·cm^−3^·K^−1^)	*σ* × 10^6^/(g·cm^−3^)	*R* ^2^
Men-OctA (1:2)	1.13092	−7.645	6.52	0.9999
Men-DecA (1:1)	1.11497	−7.341	8.41	0.9999
Thy-but (1:1)	1.15447	−8.098	1.58	0.9994
Thy-hex (1:1)	1.13753	−7.826	1.02	0.9997
Thy-OctA(1:1)	1.17943	−8.054	9.75	0.9998
Thy-OleA (1:1)	1.13396	−7.346	1.10	0.9996
TBPBr-OctA (1:1)	1.21198	−6.718	4.48	0.9999
TBPBr-LauA (1:1)	1.19213	−6.782	1.02	0.9996

**Table 2:** Molar masses (*M*), molar volumes (*V*_m_) and relative molar volume changes (Δ*V*_m_) of the investigated ESSs in the temperature range 293.15–313.15 K.

System	*M* (g∙mol^−1^)	*V*_m_ (cm^3^∙mol^−1^)					Δ*V*_m_ (%)
*T* (K)		293.15	298.15	303.15	308.15	313.15	293.15–313.15 K
Men-OctA (1:2)	148.23	163.47	164.16	164.86	165.56	166.27	1.72
Men-DecA (1:1)	164.27	182.57	183.32	184.07	184.83	185.60	1.66
Thy-but (1:1)	112.17	122.33	122.85	123.39	123.94	124.54	1.80
Thy-hex (1:1)	126.20	138.98	139.56	140.17	140.78	141.42	1.75
Thy-OctA (1:1)	147.22	156.08	156.72	157.39	158.08	158.79	1.74
Thy-OleA (1:1)	216.35	235.54	236.44	237.39	238.36	239.37	1.62
TBPBr-OctA (1:1)	241.78	238.20	238.97	239.77	240.58	241.39	1.34
TBPBr-LauA (1:1)	269.83	271.68	272.57	273.49	274.45	275.44	1.38

Standard uncertainty is: *u*(*V*_m_) ≤ 0.0015 cm^3^·mol^−1^.

**Table 3:** Viscosity values (*η*) of various eutectic solvent mixtures in the temperature range from 293.15 to 313.15 K at atmospheric pressure (*p* = 0.1 MPa).

System	*η* (mPa∙s)					Δ*η* (%)
*T*/K	293.15	298.15	303.15	308.15	313.15	293.15–313.15 K
Men-OctA (1:2)	10.48	8.631	7.202	6.081	5.191	50.5
Men-DecA (1:1)	21.38	16.56	13.08	10.51	8.572	59.9
Thy-but (1:1)	7.601	6.152	5.103	4.302	3.601	52.6
Thy-hex (1:1)	7.701	5.853	4.452	3.301	2.451	68.2
Thy-OctA (1:1)	11.35	9.151	7.650	6.351	5.254	53.7
Thy-OleA (1:1)	35.53	23.52	15.47	9.058	4.401	87.6
TBPBr-OctA (1:1)	455.9	335.9	254.9	190.5	144.0	68.4
TBPBr-LauA (1:1)	562.4	413.9	310.4	235.5	180.0	68.0

Relative standard uncertainties are: *u*_r_(*η*) = 0.5% for Lovis 2000 M/ME and *u*_r_(*η*) = 1% of the range in use for Brookfield DV-II+ Pro; *u*(*T*) = 0.02 K.

**Table 4:** Viscosity flow activation values (*E_a_*) of various eutectic solvent systems.

System	*E*_a_/(kJ·mol^−1^)	*σ/*(ln(mPa∙s))	*R* ^2^
Men-OctA (1:2)	26.81	0.007	0.99956
Men-DecA (1:1)	34.87	0.010	0.99938
Thy-but (1:1)	26.24	0.009	0.99935
Thy-hex (1:1)	28.28	0.022	0.99830
Thy-OctA (1:1)	29.11	0.008	0.99948
Thy-OleA (1:1)	38.21	0.136	0.97942
TBPBr-OctA (1:1)	43.85	0.008	0.99979
TBPBr-LauA (1:1)	43.40	0.004	0.99995

**Table 5:** Vogel–Fulcher–Tammann (VFT) fitting parameters for the viscosity–temperature correlation of the investigated ESSs, with regression statistics and the calculated activation energy at *T =* 298.15 K, *E*_η(298.15K)_.

System	*A*	*B*	*T*_o_/K	*σ/*(ln(mPa∙s))	*R* ^2^	*E*_η(298.15K)_/kJ∙mol^−1^
Men-OctA (1:2)	−3.15	750	156	6.06 × 10^−5^	0.9999	27.43
Men-DecA (1:1)	−3.48	807	170	2.26 × 10^−4^	0.9999	36.32
Thy-but (1:1)	−3.88	823	153	6.65 × 10^−3^	0.9997	28.87
Thy-hex (1:1)	−3.46	864	173	3.67 × 10^−3^	0.9995	30.77
Thy-OctA (1:1)	−2.99	642	171	8.23 × 10^−3^	0.9994	29.35
Thy-OleA (1:1)	−3.42	1065	138	3.47 × 10^−3^	0.9997	30.69
TBPBr-OctA (1:1)	−6.58	2732	66	1.05 × 10^−3^	0.9997	37.46
TBPBr-LauA (1:1)	−7.10	2905	77	2.11 × 10^−3^	0.9999	43.90

**Table 6:** Electrical conductivity (*κ*) of ESSs at atmospheric pressure (*p* = 0.1 MPa) over the temperature range from 293.15 to 313.15 K.

SYSTEM	*κ*/(μS·cm^−1^)				
*T*/K	293.15	298.15	303.15	308.15	313.15
Men-OctA (1:2)	0.151	0.153	0.157	0.159	0.163
Men-DecA (1:1)	0.152	0.153	0.156	0.159	0.162
Thy-but (1:1)	0.002	0.003	0.003	0.004	0.006
Thy-hex (1:1)	0.004	0.006	0.007	0.008	0.012
Thy-OctA (1:1)	0.351	0.354	0.387	0.452	0.567
Thy-OleA (1:1)	0.252	0.266	0.287	0.321	0.415
TBPBr-OctA (1:1)	116.3	138.2	173.8	216.9	264.2
TBPBr-LauA (1:1)	71.06	86.02	107.0	133.5	163.9

Standard uncertainty is: *u*_r_(*κ*) = 0.5%.

**Table 7:** Molar conductivity (*Λ*) of ESSs at atmospheric pressure (*p* = 0.1 MPa) over the temperature range from 293.15 to 313.15 K.

SYSTEM	*Λ*∙10^5^ (S·cm^2^·mol^−1^)				
*T*/K	293.15	298.15	303.15	308.15	313.15
Men-OctA (1:2)	2.468	2.512	2.588	2.632	2.710
Men-DecA (1:1)	2.775	2.805	2.871	2.939	3.007
Thy-but (1:1)	0.02447	0.03685	0.03702	0.04958	0.07472
Thy- hex (1:1)	0.05559	0.08374	0.09812	0.1126	0.1697
Thy-OctA (1:1)	5.478	5.548	6.091	7.145	9.003
Thy-OleA (1:1)	5.936	6.289	6.813	7.651	9.934
TBPBr-OctA (1:1)	2770	3303	4167	5218	6378
TBPBr-LauA(1:1)	1931	2345	2926	3664	4514

**Table 8:** Ionicity (%) of the investigated ESSs calculated from Equations (10) and (11) in the temperature range 293.15–313.15 K at *p* = 0.1 MPa.

SYSTEM	IONICITY (%)				
*T*/K	293.15	298.15	303.15	308.15	313.15
Men-OctA (1:2)	0.000259	0.000217	0.000186	0.000160	0.000141
Men-DecA (1:1)	0.000593	0.000464	0.000376	0.000309	0.000258
Thy-but (1:1)	0.00000186	0.00000227	0.00000189	0.00000213	0.00000269
Thy- hex (1:1)	0.00000428	0.00000490	0.00000437	0.00000372	0.00000416
Thy-OctA (1:1)	0.000622	0.000508	0.000466	0.000454	0.000473
Thy-OleA (1:1)	0.002109	0.001479	0.001054	0.000692	0.000437
TBPBr-OctA (1:1)	12.63	11.09	10.62	9.94	9.18
TBPBr-LauA (1:1)	10.86	9.70	9.08	8.63	8.13

**Table 10:** Compounds used in this study and their main specifications.

Compound	Chemical Structure	Abbreviation	Supplier	CAS Number	Purity (wt%)
Tetrabutylphosphonium bromide	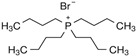	TBPBr	Sigma-Aldrich	3115-68-2	>98
Thymol	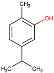	Thy	Sigma-Aldrich	89-83-8	>98.5
DL-Menthol	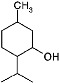	Men	Sigma-Aldrich	89-78-1	>98
Butanol	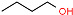	but	Sigma-Aldrich	71-36-3	>99.8
Hexanol	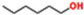	hex	Sigma-Aldrich	111-27-3	>99
Octanoic acid	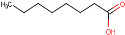	OctA	Sigma-Aldrich	124-07-2	>99
Decanoic acid	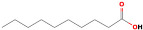	DecA	Sigma-Aldrich	334-48-5	>99.5
Lauric Acid	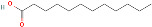	LauA	Sigma-Aldrich	143-07-7	>98
Oleic acid	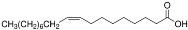	OleA	Sigma-Aldrich	112-80-1	>99

**Table 11:** Composition and abbreviations of prepared ESSs.

Component 1	Component 2	Abbreviations	Molar Ratio
Menthol	Octanoic Acid	Men-OctA	1:2
	Decanoic Acid	Men -DecA	1:1
Thymol	Butanol	Thy-but	1:1
	Hexanol	Thy-hex	1:1
	Octanoic Acid	Thy-OctA	1:1
	Oleic Acid	Thy-OleA	1:1
Tetrabutylphosphonium bromide (TBPBr)	Octanoic Acid	TBPBr-OctA	1:1
	Lauric Acid	TBPBr-LauA	1:1

## Data Availability

The original contributions presented in this study are included in the article. Further inquiries can be directed to the corresponding authors.
